# The influence of stimulus and behavioral histories on predictive control of smooth pursuit eye movements

**DOI:** 10.1038/s41598-021-01733-1

**Published:** 2021-11-16

**Authors:** Takeshi Miyamoto, Yutaka Hirata, Akira Katoh, Kenichiro Miura, Seiji Ono

**Affiliations:** 1 Faculty of Health and Sport Sciences, University of Tsukuba, Ibaraki, Japan; 2 Department of Robotic Science and Technology, Chubu University College of Engineering, Kasugai, Japan; 3 Center for Mathematical Science and Artificial Intelligence, Chubu University, Kasugai, Japan; 4 Academy of Emerging Sciences, Chubu University, Kasugai, Japan; 5 Department of Physiology, Tokai University School of Medicine, Kanagawa, Japan; 6 Department of Pathology of Mental Diseases, National Institute of Mental Health, National Center of Neurology and Psychiatry, Tokyo, Japan; 7 Graduate School of Medicine, Kyoto University, Kyoto, Japan

**Keywords:** Motor control, Visual system

## Abstract

The smooth pursuit system has the ability to perform predictive feedforward control of eye movements. This study attempted to examine how stimulus and behavioral histories of past trials affect the control of predictive pursuit of target motion with randomized velocities. We used sequential ramp stimuli where the rightward velocity was fixed at 16 deg/s while the leftward velocity was either fixed (predictable) at one of seven velocities (4, 8, 12, 16, 20, 24, or 28 deg/s) or randomized (unpredictable). As a result, predictive pursuit responses were observed not only in the predictable condition but also in the unpredictable condition. Linear mixed-effects (LME) models showed that both stimulus and behavioral histories of the previous two or three trials influenced the predictive pursuit responses in the unpredictable condition. Intriguingly, the goodness of fit of the LME model was improved when both historical effects were fitted simultaneously rather than when each type of historical data was fitted alone. Our results suggest that predictive pursuit systems allow us to track randomized target motion using weighted averaging of the information of target velocity (stimulus) and motor output (behavior) in past time sequences.

## Introduction

Smooth pursuit eye movements allow us to continuously follow a moving object on or near the line of sight at the center of the fovea, resulting in high visual acuity for the target. In general, there is an approximately 100 ms gap from the onset of target motion to the onset of eye movement in the human oculomotor system^1,2^, and this delay can lead to a loss of visual information because visual acuity declines as the target moves from the fovea to that of the retinal periphery^3^. To overcome the inherent delay, the oculomotor system has the ability to perform predictive feedforward control of eye movements^4,5^. When the timing of onset/offset or directional changes of the target motion is predictable, preceding eye velocity is generated according to future visual motion^6,7^. Such predictive pursuit responses are thought to be preprogrammed^8,9^, and their timing and scale can be built up with repeated trials^8,10,11^.

Predictive pursuit is known to occur even when the timing of onset/offset or directional change of visual motion is randomized, and the timing of onset of the predictive pursuit is influenced by the history of previous trials^12,13^. Several studies have demonstrated that the effect of stimulus history on the onset of predictive pursuit does not depend solely on the most recent trial but is determined by the sequence of several previous trials, similar to the branches of a tree^14,15^. Moreover, other studies have demonstrated that the timing of onset of predictive pursuit to visual motion with the randomized timing of directional changes is quantitatively associated with the ramp duration of previous trials, and the influence of previous trials on predictive pursuit declines progressively as the previous trial progresses further into the past^16–18^. Taken together, the stimulus information of previous trials appears to be continuously averaged and used to determine the timing of the onset of predictive pursuit to visual motion with randomized timing.

In addition to the timing of pursuit eye movement onset, eye velocity is an important component of predictive smooth pursuit because errors between eye and target velocity can cause the image of the moving target to be blurred. Although predictive eye velocity is regulated according to the predictable velocity of future target motion^12,19,20^, it is still uncertain how past trials affect the control of predictive pursuit to target motion with randomized velocity. For predictive pursuit of randomly timed stimuli, a model of ocular pursuit described by previous studies has proposed that a continual estimation of the timing of onset of future target motion is constructed by weighted averaging of stimulus history and retained in working memory so that predictive pursuit can be initiated^6,16^. This model is supported by fMRI studies that show the activity of the dorsolateral prefrontal cortex (DLPFC), which is associated with working memory^21^, during smooth pursuit of predictable target motion^22,23^. Considering the behavioral evidence that the velocity of predictive pursuit accumulates over repeated trials^8,10,11^, target velocity information may also be retained in working memory. However, unlike onset timing, the velocity of predictive pursuit can also be modulated by internal gain. The frontal eye field (FEF), in particular, the frontal pursuit area (FPA), is known to play a role in the control of internal gain^24^. A recent study demonstrated that the control of the gain in the FPA is modulated by a Bayesian inference, in which the interaction between priors based on past experience and current sensory evidence creates a posterior distribution regarding target velocity that could regulate the smooth pursuit gain in the initial phase^25^. Moreover, a study focusing on eye motion in response to perturbations of target motion has demonstrated that the eye velocity induced by perturbations is greater when the previous trial entailed smooth pursuit than when it involved fixation^26^. Although the above studies are not about predictive pursuit, they suggested that behavioral history influences the eye velocity of predictive pursuit. Therefore, we hypothesized that stimulus (i.e., velocity of target motion) and behavioral (i.e., pursuit responses) histories affect the eye velocity of predictive pursuit. This study was designed to clarify, quantitatively, the influence of both stimulus and behavioral histories on the predictive pursuit of visual motion with randomized velocity.

## Materials and methods

### Observers

The observers were 12 adults (3 women and 9 men, mean age: 23.6 [SD: 1.3] years old), and they reported having normal or corrected-to-normal vision and no known visuomotor deficits. The observers were diagnosed with neither stereoscopic problems nor strabismus. All observers gave written informed consent in accordance with the Declaration of Helsinki. All protocols were approved by the Research Ethics Committee at the Faculty of Health and Sport Sciences, University of Tsukuba.

### Apparatus

The observers sat 57 cm in front of a CRT monitor (22-inch, RDF223G, Mitsubishi Electric Co., Tokyo, Japan, refresh rate: 60 Hz, spatial resolution: 800 × 600 pixels) with head stabilized by a chin rest and a forehead restraint. Eye movements from the right eye were detected using a video-based eye tracking system. In this system, eye position signals were detected by reflections of the infrared light on the cornea and a black image of the pupil was captured by an infrared camera (GS3-U3-41C6NIR, FLIR systems Inc., OR, USA)^27^. The eye position signals detected by the system were digitized at 1 kHz with 16-bit precision using CED-Micro 1401 hardware (Cambridge Electronic Designs, Cambridge, England). Prior to the task, the eye position signals were calibrated by requiring the observers to fixate on a target spot (diameter of 0.3 deg) at known horizontal and vertical eccentricities on a uniform black background in a binocular viewing condition. All the visual stimuli used in the calibration and experimental tasks were generated by Psychophysics Toolbox extensions on MATLAB (MathWorks, MA, USA).

### Stimulus and procedure

**Figure 1:**
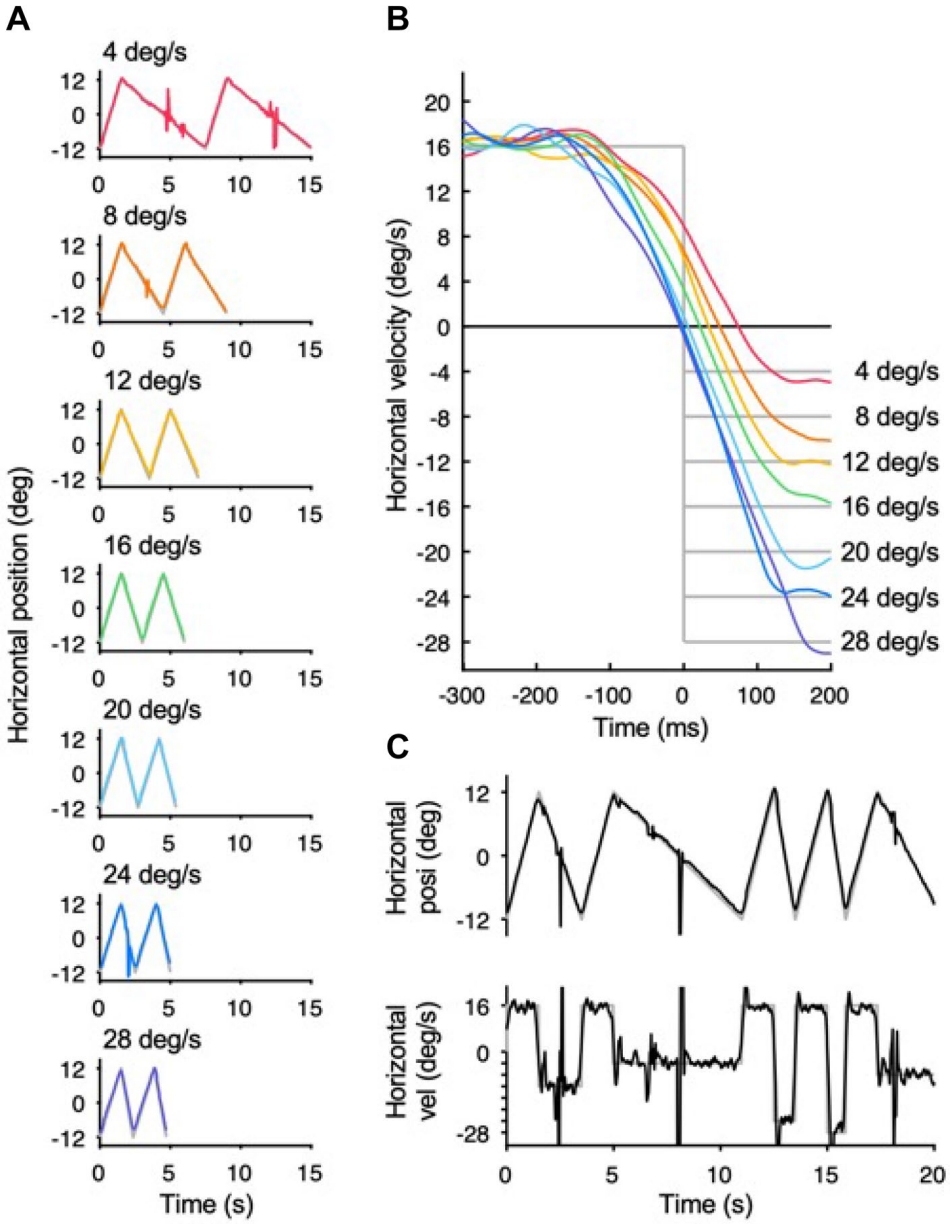
(A) Examples of eye position traces (colored lines) from a representative observer in the predictable condition where the rightward velocity was fixed at 16 deg/s while the leftward velocity was fixed at one of seven velocities (4, 8, 12, 16, 20, 24, or 28 deg/s). One trial consisted of a sequence of the rightward and leftward ramp motion, and each panel shows a typical example of two consecutive trials. There was no pause between ramps, and the starting position was always the same for the initial rightward ramp. Gray lines represent the target position. B: Averaged traces of eye velocity from a representative observer in the predictable condition. The colors of each eye velocity trace correspond to the seven target velocities (same as in Fig. 1A), and gray lines represent target velocities. The time of 0 ms represents the timing of target reversal. C: Examples of eye position (upper panel) and velocity (lower panel) transition from a representative observer in the unpredictable condition where the leftward velocity was randomized within the seven velocities. The figure includes a typical example of five consecutive trials. Black lines represent the eye position and velocity whereas gray lines represent target motion. In all the panels, upward deflections show rightward eye motion.

A target consisting of a white Gaussian dot (SD: 0.15 deg, luminance: 70 cd/m^2^) on a uniform black background (luminance: 0.1 cd/m^2^) moved horizontally with a constant velocity over a distance of 24 deg. White downward arrows (0.5 deg × 1.0 deg) were always presented at 1 deg above each end of the travel distance to inform the observers of the target's reversal position. One trial consisted of a sequence of rightward and leftward ramp motions. In this sequence, the rightward velocity was fixed at 16 deg/s while the leftward velocity was either fixed (predictable condition) at one of seven velocities (4, 8, 12, 16, 20, 24, or 28 deg/s) or randomly presented at one of the seven velocities (unpredictable condition). There was no pause between ramps, and the starting position was always the same for the initial rightward ramp (i.e., the trajectory of the target was an irregular triangular waveform). The observers were instructed to track the target as accurately as possible. In the predictable condition (Fig. 1A), one of the seven velocities was repeated 30 times within a block, the order of the seven velocities was randomized, and there was a one-minute interval between each block. In the unpredictable condition (Fig. 1C), the seven velocities were presented randomly within one block. Each block in the unpredictable condition consisted of 49 trials (7 velocities × 7 trials), and 5 blocks were conducted with one-minute intervals between blocks. The order of conditions was counterbalanced among the observers. Before the start of each block, the observers were informed of the condition that would be used in the next block; if the next block was the predictable condition, observers were informed of the target velocity in advance. Prior to the task, the observers practiced the procedure by performing 21 trials following the presentation method of each condition.

### Eye movement recordings and analysis

**Figure 2:**
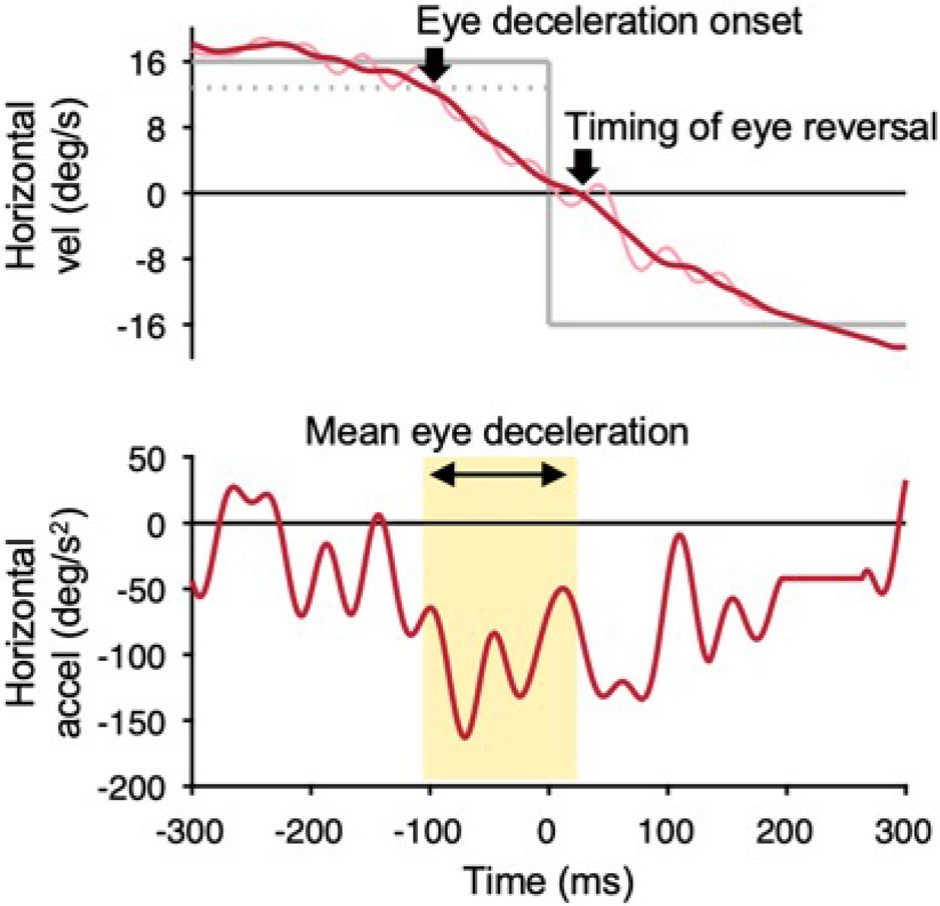
Eye velocity in a representative trial when the target speed was 16 deg/s. The top panel shows the eye velocity after filtering (30 Hz low-pass filter, light red line), the eye velocity with moving average applied to it (40-ms window, dark red line), and the target velocity (gray solid line). The threshold to detect the onset of predictive pursuit is represented by a gray dotted line. The bottom panel shows eye acceleration in the same trial. The yellow shaded region represents the time that the mean eye deceleration was calculated (i.e., the time between the eye deceleration onset and timing of eye reversal). The time of 0 ms represents the timing of target reversal. Upward deflections show rightward eye motion.

Eye velocity and acceleration were generated by digital differentiation of the position arrays using the central difference algorithm in MATLAB (MathWorks). Velocity and acceleration data were filtered using an 80-point finite impulse response (FIR) digital filter with a 30 Hz passband. Saccades were identified according to a criterion of acceleration of 1000 deg/s, and linear interpolation was used to fill the gaps left by the saccades that were removed. Because oscillatory fluctuations remained in the eye velocity traces after the above filtering, a moving average was applied over a 40 ms window (Fig. 2).

The predictive pursuit for each trial was evaluated when the target reversed from right to left. First, the eye deceleration onset was defined as the timing when the eye velocity fell below a threshold calculated as 80% of the average of the eye velocities of all the trials in the steady-state phase (700–500 ms before the target reversal). Second, the timing of eye reversal was defined as the timing when the eye velocity fell below 0 deg/s. Third, the mean eye deceleration was calculated as the average eye acceleration between eye deceleration onset and the timing of eye reversal. The above evaluation methods for predictive pursuit are summarized in Fig. 2.

Trials in which the eye deceleration onset was earlier than − 300 ms relative to the timing of target reversal were excluded because most of these excluded trials involved a preceding saccade to the position where the target reverses. In addition, trials in which the timing of eye reversal occurred after 100 ms of target reversal were excluded because they were considered to have integrated feedforward signals and visual feedback. On average across all observers, 92.2% (SD: 4.4; range 82.4–94.2%) of the trials for the predictable condition and 93.9% (SD: 4.8; range 84.1–100%) for the unpredictable condition were included in the analyses.

## Results

### Predictable condition

**Figure 3:**
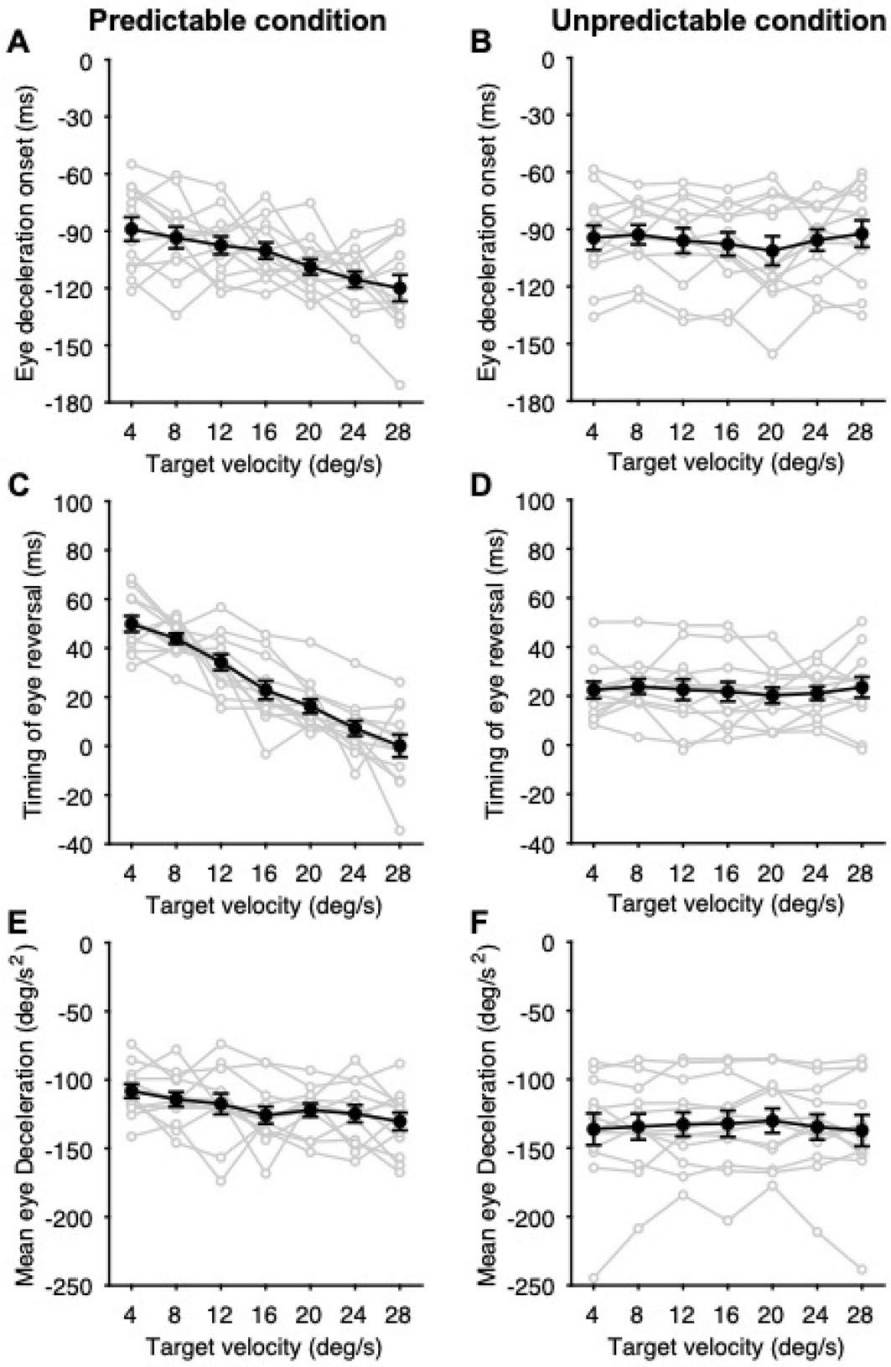
Predictive pursuit responses for the predictable condition (left panels) and unpredictable condition (right panels). (**A**,**B**) Eye deceleration onset relative to the target reversal as a function of target velocity. (**C**,**D**) Timing of eye reversal relative to the target reversal as a function of target velocity. (**E,F**) Mean eye deceleration as a function of target velocity. The gray circles and lines represent the values of each observer and black circles and lines represent the mean values of all the observers. Error bars indicate 1 SEM.

Figure 1A,B show traces of eye position and averaged eye velocity from a representative observer in the predictable condition, respectively. The eye velocity started to decay prior to the target reversal, indicating that the observer performed predictive pursuit for the future target motion. One-way repeated-measures analyses of variance (ANOVA) with the target velocity in the left direction as the independent variable showed that all of the eye deceleration onset, the timing of eye reversal, and the mean eye deceleration varied according to the target velocity (eye deceleration onset: *F*_6,66_ = 8.71, *p* = 5.48 × 10^–7^, partial *η*^2^ = 0.44, Fig. 3A; timing of eye reversal: *F*_6,66_ = 50.85, *p* = 7.27 × 10^–23^, partial *η*^2^ = 0.82, Fig. 3C; mean eye deceleration: *F*_6,66_ = 2.98, *p* = 1.23 × 10^–2^, partial *η*^2^ = 0.21, Fig. 3E). Moreover, the within-subjects correlation coefficient, which is a method used to focus on the changes in variables within each observer^28^, showed that the timing of eye reversal was closely associated with the target velocity (*r* = − 0.90, *p* = 5.30 × 10^–28^) compared to eye deceleration onset (*r* = − 0.66, *p* = 2.43 × 10^–10^) and mean eye deceleration (*r* = − 0.43, *p* = 1.35 × 10^–4^). Therefore, we used the timing of eye reversal as a representative index of predictive pursuit to examine the effect of past trials on the current trial.

**Figure 4:**
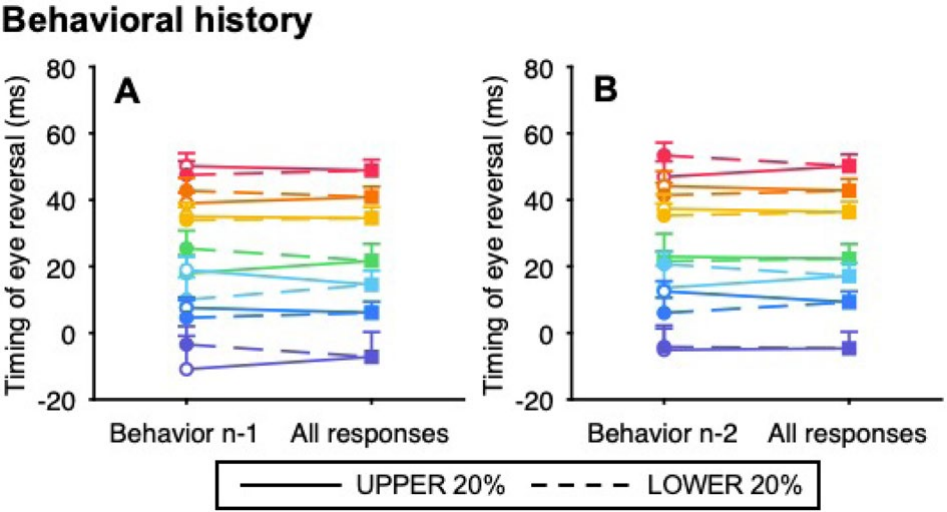
Effects of behavioral history on the timing of eye reversal in the predictable condition. The timing of eye reversal was ranked in ascending order at each target velocity, with the top 20% classified as “UPPER” and the bottom 20% classified as “LOWER”. Then, trials were sorted based on the classification of the n-1 (**A**) or n-2 trial (**B**). The left node shows the mean timing of eye reversal sorted by the classification, and the right node shows the mean of both trials. In the left node, the open symbols and solid lines represent the trials preceded by a response from the UPPER trials, and the filled symbols and dashed lines represent the trials preceded by a response from the LOWER trials. The color of symbols and lines correspond to the seven target velocities (same as in Fig. 1A). Error bars indicate 1 SEM.

In the predictable condition, we tested the effect of behavioral history alone on the timing of eye reversal because the stimulus (leftward target velocity) was constant within a block. Several studies have examined the quantitative effect of past trials on the predictive pursuit response in the current trial (n) using linear regression analysis with n-back trials as the independent variable^16–18^. However, the effect of multicollinearity could not be discounted because the behavioral history of up to 5 back trials (i.e., the timing of eye reversal back to the last 5 trials) contained more than moderate positive correlations in at least one combination between n-back trials for all observers. Therefore, we tested the effect of behavioral history on the predictive pursuit response using the node (or tree) method, where trials were grouped based on the characteristics of previous trials^12–15,17,29^. The timing of eye reversal was ranked in ascending order at each target velocity for each observer, with the top (earlier) 20% classified as “UPPER” and the bottom (later) 20% classified as “LOWER”. Then, trials were sorted based on the classification of the n-1 (Fig. 4A) or n-2 trial (Fig. 4B). For both classifications based on the n-1 and n-2 trials, two-way ANOVA (target velocity x classification based on the previous trial) revealed neither a main effect of the classification (n-1 trial: *F*_1,11_ = 0.07, *p* > 0.99, partial *η*^2^ = 0.04; n-2 trial: *F*_1,11_ = 0.04, *p* > 0.99, partial *η*^2^ < 0.01) nor an interaction (n-1 trial: *F*_6,66_ = 1.28, *p* = 0.28, partial *η*^2^ = 0.10; n-2 trial: *F*_6,66_ = 0.66, *p* = 0.68, partial *η*^2^ = 0.06).

### Unpredictable condition

Predictive pursuit responses were observed even in the unpredictable condition (Fig. 1C), but as a matter of course, they did not correlate with the future target velocity (eye deceleration onset: *F*_6,66_ = 1.38, *p* = 0.24, partial *η*^2^ = 0.11, *r* = 0.04, Fig. 3B; timing of eye reversal: *F*_6,66_ = 0.56, *p* = 0.76, partial *η*^2^ = 0.05, *r* = 0.06, Fig. 3D; mean eye deceleration: *F*_6,66_ = 0.55, *p* = 0.77, partial *η*^2^ = 0.05, *r* = 0.01, Fig. 3F).

**Figure 5:**
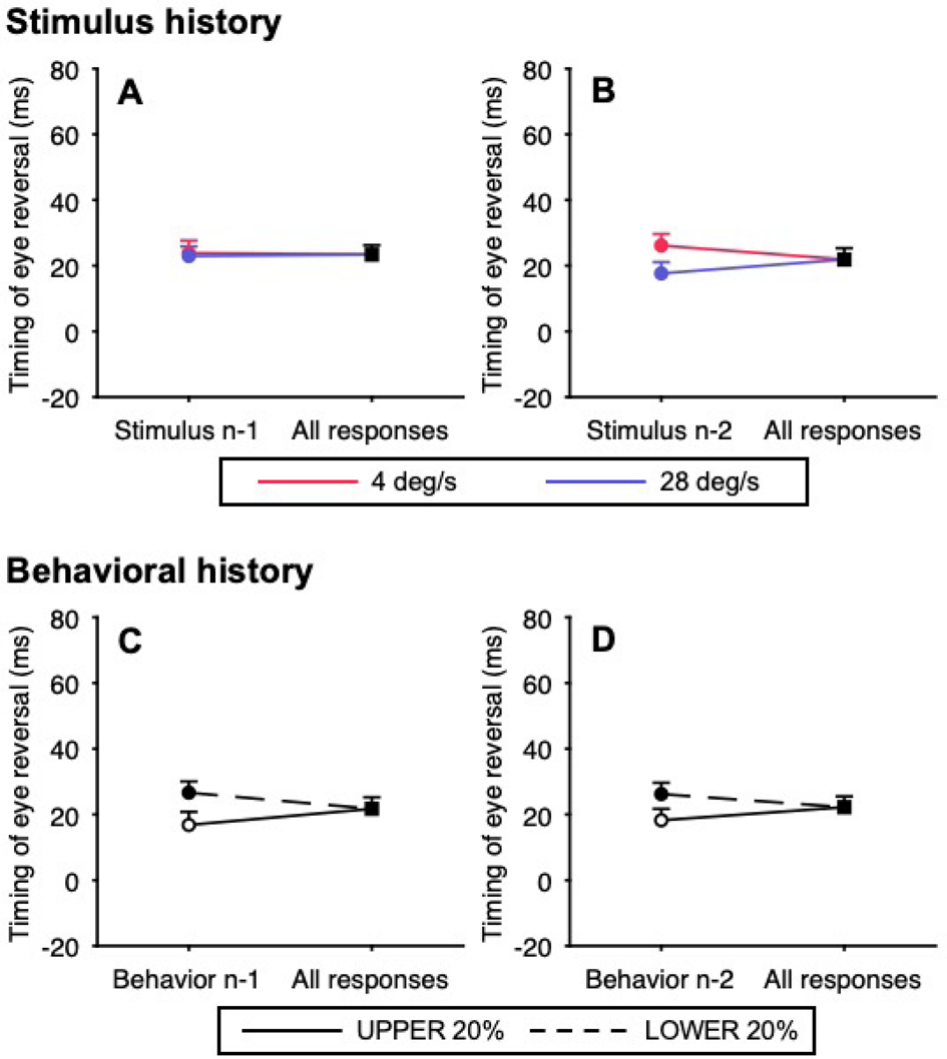
Effects of stimulus and behavioral histories on the timing of eye reversal in the unpredictable condition. For the stimulus history, trials were sorted based on whether the target velocity in the n-1 (**A**) or n-2 trial (**B**) was 4 deg/s (red) or 28 deg/s (blue). For the behavioral history, trials were sorted by the same classification as the predictable condition (**C**: trials sorted by the n-1 trial; **D**: trials sorted by the n-2 trial). In the left node, the open symbol and solid line represent the trials preceded by a response from the UPPER trials, and the filled symbols and dushed lines represent the trials preceded by a response from the LOWER trials. Error bars indicate 1 SEM.

The effects of stimulus and behavioral histories on predictive pursuit were examined using the timing of eye reversal, as with the predictable condition. First, to test the effect of each history alone on the predictive pursuit response, we used the node method in the unpredictable condition. For the stimulus history, trials were sorted based on whether the target velocity in the n-1 or n-2 trial was 4 deg/s (slowest) or 28 deg/s (fastest). For the behavioral history, trials were sorted by the same classification as the predictable condition. An effect of stimulus history was found when trials were sorted based on the n-2 trial (*t*_*11*_ = 3.61, *p* = 4.10 × 10^–3^, Cohen’s *d* = 1.09, Fig. 5B) but not when they were sorted based on the n-1 trial (*t*_*11*_ = 0.24, *p* = 0.81, Cohen’s *d* = 0.07, Fig. 5A). In contrast, an effect of behavioral history was found when trials were sorted based on both the n-1 trial (*t*_*11*_ = 4.09, *p* = 1.79 × 10^–3^, Cohen’s *d* = 1.23, Fig. 5C) and the n-2 trial (*t*_*11*_ = 3.75, *p* = 3.23 × 10^–3^, Cohen’s *d* = 1.13, Fig. 5D).

Then, to examine the quantitative effect of stimulus and behavioral histories on predictive pursuit, we fitted linear mixed-effects (LME) models with the timing of eye reversal in the current trial as the dependent variable, the target velocity (stimulus) and the timing of eye reversal (behavior) back to the n-5 trial as the independent variables, and the individual intercept as the random effect (performed in MATLAB with the “fitlme” function in the Statistics and Machine Learning Toolbox. The significance level of fixed effects was set at 0.05). When fitting the LME models using the n-5 trial, since it was not possible to calculate the historical effect of the first five trials of each block, data from the first 5 trials were used in the analysis as the history effect of the sixth and subsequent trials (independent variables) but not as the dependent variable. A total of 15 LME models were created, including those in which each history alone was incorporated into the model and those in which both types of historical data were incorporated (each with varying length, going back from the n-1 to the n-5 trials). Based on Akaike’s information criterion (AIC), the model that incorporated the stimulus and behavioral histories back to the n-5 trial was adopted (Model 15 in Table 1). In the model, significant fixed effects were found for the stimulus history of the n-2 (estimate ± SE = − 0.417 ± 0.082, *t*_*2411*_ = 5.11, *p* = 3.53 × 10^–7^) and n-3 trials (estimate ± SE = − 0.215 ± 0.082, *t*_*2411*_ = 2.64, *p* = 8.41 × 10^–3^) and for the behavioral history of the n-1 (estimate ± SE = 0.078 ± 0.018, *t*_*2411*_ = 4.26, *p* = 2.09 × 10^–5^) and n-2 trials (estimate ± SE = 0.093 ± 0.018, *t*_*2411*_ = 5.16, *p* = 2.64 × 10^–7^). Note that these independent variables, which showed significant fixed effects in Model 15, were also found to be significant in the models that were not adopted. As a side note, the correlation coefficients for all combinations of 10 independent variables calculated per observer ranged from -0.28 to 0.25, and the values of the variance inflation factor (VIF) for each independent variable in Model 15 were less than 1.17. The effect of multicollinearity on the coefficients was determined to be negligible.

**Table 1:** Linear mixed-effects models with the timing of eye reversal in the current trial (n) in the unpredictable condition.

Variable	Model 1		Model 2		Model 3		Model 4		Model 5	
	Estimate	*SE*	Estimate	*SE*	Estimate	*SE*	Estimate	*SE*	Estimate	*SE*
(Intercept)	23.272	3.047	30.146	3.415	34.514	3.770	35.766	4.086	37.936	4.349
Stimulus (n−1)	− 0.026	0.078	− 0.099	0.079	− 0.104	0.079	− 0.102	0.079	− 0.101	0.079
Stimulus (n−2)			− **0.356**	**0.080**	− **0.406**	**0.082**	− **0.408**	**0.082**	− **0.403**	**0.082**
Stimulus (n−3)					− **0.216**	**0.080**	− **0.231**	**0.082**	− **0.233**	**0.082**
Stimulus (n−4)							− 0.063	0.080	− 0.087	0.081
Stimulus (n−5)									− 0.116	0.080
AIC	23,897.807		23,880.119		23,874.762		23,876.132		23,876.018	

Variable	**Model 6**		**Model 7**		**Model 8**		**Model 9**		**Model 10**	
	Estimate	*SE*	Estimate	*SE*	Estimate	*SE*	Estimate	*SE*	Estimate	*SE*
(Intercept)	20.628	2.524	18.505	2.273	17.709	2.197	17.492	2.196	17.033	2.167
Behavior (n−1)	**0.094**	**0.018**	**0.081**	**0.018**	**0.083**	**0.018**	**0.081**	**0.018**	**0.080**	**0.018**
Behavior (n−2)			**0.098**	**0.017**	**0.094**	**0.018**	**0.094**	**0.018**	**0.093**	**0.018**
Behavior (n−3)					**0.036**	**0.018**	0.034	0.018	0.030	0.018
Behavior (n−4)							0.015	0.018	0.012	0.018
Behavior (n−5)									0.031	0.018
AIC	23,784.235		23,700.346		23,632.288		23,575.456		23,521.373	

Variable	Model 11		Model 12		Model 13		Model 14		Model 15	
	Estimate	*SE*	Estimate	*SE*	Estimate	*SE*	Estimate	*SE*	Estimate	*SE*
(Intercept)	21.007	2.807	26.116	2.996	29.546	3.359	29.483	3.742	30.620	4.041
Stimulus (n−1)	− 0.024	0.078	− 0.101	0.079	− 0.102	0.079	− 0.095	0.079	− 0.096	0.079
Stimulus (n−2)			− **0.375**	**0.079**	− **0.428**	**0.081**	− **0.423**	**0.082**	− **0.417**	**0.079**
Stimulus (n−3)					− **0.207**	**0.079**	− **0.213**	**0.081**	− **0.215**	**0.082**
Stimulus (n−4)							0.011	0.079	0.034	0.081
Stimulus (n−5)									− 0.074	0.080
Behavior (n−1)	**0.094**	**0.018**	**0.081**	**0.018**	**0.080**	**0.018**	**0.078**	**0.018**	**0.078**	**0.018**
Behavior (n−2)			**0.099**	**0.017**	**0.096**	**0.017**	**0.096**	**0.018**	**0.093**	**0.018**
Behavior (n−3)					0.036	0.018	0.035	0.018	0.029	0.018
Behavior (n−4)							0.012	0.018	0.001	0.018
Behavior (n−5)									0.029	0.018
AIC	23,786.139		23,682.016		23,608.671		23,554.418		23,501.849	
Dependent variable: the timing of eye reversal in the current trial										
Significant fixed effects are in bold (*p* < 0.05)										
Model 15 (including stimulus and behavioral histories back to the n-5 trial: the shaded area) is adopted based on the AIC										

**Figure 6:**
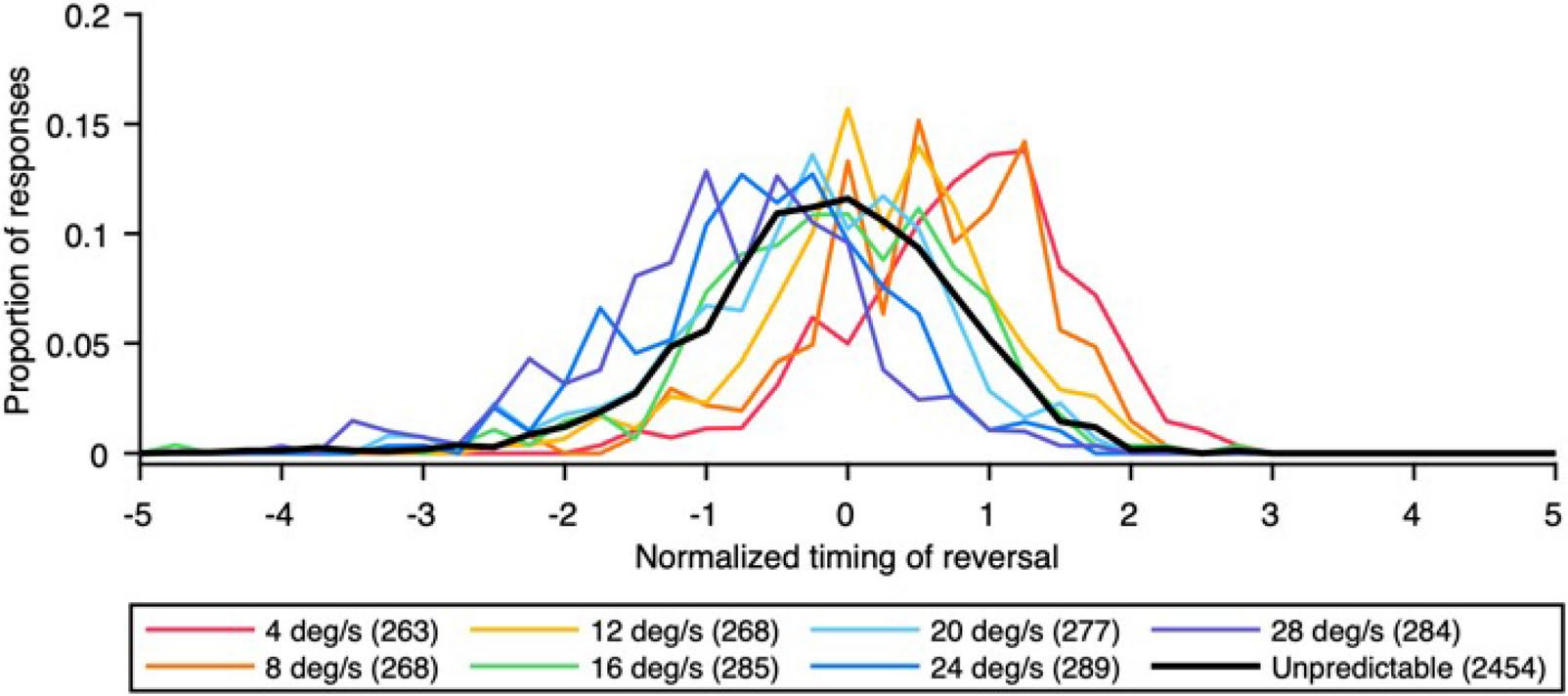
Pooled distribution of the normalized timing of eye reversal across all observers (bin size, 0.25). The values of median of each distribution are below; (4 deg/s: 1.00; 8 deg/s: 0.71; 12 deg/s: 0.39; 16 deg/s: 0.06; 20 deg/s: -0.08; 24 deg/s: − 0.44; 28 deg/s: − 0.68; unpredictable: 0.02). The colored and black lines indicate the predictable and unpredictable conditions, respectively. The values in parentheses in the legend represent the number of trials included in each distribution.

The above node method and LME model analyses showed that the predictive pursuit response was influenced by both stimulus and behavioral histories, but it is still unclear how the information of past trials determines the predictive pursuit response. Previous studies have argued two possibilities for how information from past trials affects predictive pursuit responses. One is that predictive pursuit responses are determined by the weighted average of information from past trials^16–18^. The other is that the experience of past trials promotes the expectation of a specific target velocity, which is reflected in predictive pursuit responses (so-called a Markov model)^29^. We focused on the distribution of the timing of eye reversal to examine whether the predictive pursuit response in the unpredictable condition of this study is consistent with either of the above findings. The distribution of the predictive pursuit response would be unimodal if the information from past trials is represented as a weighted average, whereas the distribution would be multimodal if the predictive pursuit response fits the Markov model^30^. Because the distribution of the timing of eye reversal varied slightly among the observers, the values for each trial for each subject were normalized by the mean of all the trials in both conditions combined. Figure 6 shows the pooled distribution of the normalized timing of eye reversal across all observers. The distribution of the timing of eye reversal in the unpredictable condition (black line in Fig. 6) was unimodal, suggesting that the predictive pursuit response was the result of weighted averaging of the information from past trials, rather than the result of the observer's expectation of any target velocity.

## Discussion

The purpose of this study was to clarify the influences of stimulus and behavioral histories on the predictive pursuit of target motion with randomized velocity using irregular triangular target motion stimuli. In experimental trials, the target moved to the right at 16 deg/s and then reversed to the left at one of seven velocities. For the predictable condition in which the fixed target velocity to the left was repeated within a block, the predictive pursuit (deceleration of pursuit) preceded the target reversal, as was done in previous studies^8,12,19,31^. The eye deceleration onset and the mean eye deceleration varied depending on the target velocity, and the timing of eye reversal, which depends on both, was strongly correlated with the forthcoming target velocity. The node method of analysis for the timing of eye reversal showed no effect of behavioral history on the predictive pursuit response in the predictive condition. Thus, it is likely that the observers’ knowledge that the target velocity would be constant within a block suppressed the history effect on predictive tracking, as shown in previous studies that demonstrate that external cues could override the history effect^14,32^. Although predictive pursuit was observed even in the unpredictable condition in which the target velocity to the left was randomized, the responses did not correlate with the future target velocity. The predictive pursuit responses in the unpredictable condition appeared to fall within the range observed in the predictable condition, which is referred to as a centering strategy and has been reported by previous studies focusing on velocity randomization^12^ and timing randomization^17^. This outcome plausible in light of the historical effect on the predictive pursuit of target motion with randomized velocity revealed by the LME model; also, it appears to be effective in preventing large errors between eye and target motion in unpredictable situations while compensating for inherent visuomotor delays. Both the node method and LME model showed the effect of stimulus and behavioral histories on the predictive pursuit response. To be precise, the adopted LME model incorporating both stimulus and behavioral histories from the n-5 trial and those in between showed significant fixed effects for the stimulus history of the n-2 and n-3 trials (both estimates were negative values) and for the behavioral history of the n-1 and n-2 trials (both estimates were positive values). The signs of these fixed effects indicate that "the greater the target velocities were in the previous trials" and "the greater a person’s predictive pursuit responses were in the previous trials," the greater the predictive pursuit response would be in the current trial, consistent with the results of previous studies that evaluated the history effect^14,17^. The distribution of the timing of eye reversal was unimodal, suggesting that the predictive pursuit response did not reflect the observer's expectation of any target velocity but was the result of weighted averaging of information from past trials. Here, we discuss the underlying mechanisms involved in predictive pursuit of future target motion with unpredictable velocity.

Similar to predictive tracking for target motion with randomized timing^16,17^, the history effects of previous trials on predictive pursuit were observed, and the effect of each previous trial declined progressively as the trial progressed further into the past. Please note that "past" here means that a trial is far away from the current trial in a series of trials, not simply the length of time elapsed. These similar results suggest that predictive pursuit systems use a common strategy of weighted averaging of the information from previous trials to track the randomized velocity and timing of target motion. Of the retinal and extraretinal signals involved in smooth pursuit, the latter include the effects of volition, attention, and expectation, as well as the memory of the target and eye velocity^2^. Extraretinal signals can generate predictive pursuit that is inconsistent with visual feedback (retinal input) near the timing of target reversal^19^. According to a model of smooth pursuit described by previous studies, a continual estimation of the timing is constructed by weighted averaging of stimulus history and retained in working memory so that predictive pursuit can be initiated^6,16^. Up to six variations of the memory of target velocity required for predictive pursuit can be retained^8,11^ for 14 s^10^. Given that it takes several trials for the predictive eye velocity to reach a saturation level^8,10,11^, stored and continuously averaged information about the target velocity can be used to determine the scale of predictive pursuit relative to the randomized velocity.

Our findings showed that not only the stimulus history but also the behavioral history influences predictive pursuit to target motion with randomized velocity, and the goodness of fit of the LME model is improved when both are fitted simultaneously, rather than when individual historical effects are fitted alone. For predictive pursuit to target motion with randomized timing, the influence of stimulus history has been demonstrated by multiple regression analyses^16–18^ and a node method^12,13,15,29^. Additionally, one of these studies has provided evidence that predictive pursuit is influenced by stimulus history but not behavioral history^17^. On the other hand, there was a study indicating that the onset of predictive pursuit is significantly influenced by behavioral history but not stimulus history^14^. From these studies, the influence of behavioral history on the predictive pursuit of randomly timed stimuli is ambiguous, but the following evidence may contribute to an explanation of the influence of behavioral history on the predictive pursuit of randomized velocity. For example, although observers can demonstrate predictive pursuit simply after observing a moving target^33,34^, it has been shown that the velocity of predictive pursuit is greater after active pursuit than after passive observation^35^. Similar effects have been observed in eye motion to perturbations of target motion, and the eye velocity induced by perturbations was greater when smooth pursuit was performed in previous trials than when it was not^26^. Furthermore, a study demonstrated that although repetition of the same stimulus enhances predictive eye velocity, intermixing fixation trials in a trial sequence significantly decreased the predictive eye velocity^36^. These motor-priming effects have also been observed in saccadic reaction time tasks^37,38^. Taken together, the execution of smooth pursuit affects the velocity of smooth pursuit in subsequent trials, which in turn may affect the control of predictive pursuit by means other than the stimulus history. Intriguingly, while the fixed effects of the stimulus history of the n-2 and n-3 trials were significant, those of the most recent trial (n-1) were not. A similar result has been observed in the predictive pursuit of randomly timed stimuli^17^. In contrast, the fixed effects of behavioral history appeared to correspond to the newness of the memory. Such results support the hypothesis that the information of target velocity (stimulus) and motor output (behavior) in past time sequences have, at least in part, different influences on predictive pursuit.

The supplementary eye field (SEF) is known to be involved in predictive pursuit^39,40^. Indeed, electrical microstimulation in the SEF facilitates eye velocity of predictive pursuit, the degree of which becomes greater the closer the stimulus is to the onset of target motion^41^. Furthermore, when the observer has no expectation of the target's movement, electrical stimulation of the SEF does not induce predictive pursuit^41^. Moreover, adding transcranial magnetic stimulation (TMS) to the SEF at the timing of target reversal increases eye velocity in the opposite direction, but adding TMS in the middle of the cycle does not affect eye velocity^42^. Since the SEF is directly projected from the DLPFC^43^, where the target information is likely to be stored in working memory^22,23^, the SEF is considered to be essential for the execution of predictive pursuit based on the information obtained from previous trials. However, the SEF contains only a small portion of the neurons directly involved in the generation of motor responses^44,45^, indicating that the SEF plays a premotor role in predictive pursuit^39^. The FPA, the major output center of smooth pursuit, receives signals from the SEF and generates predictive pursuit^2,6^. Unlike TMS applied to the SEF, TMS applied to the FPA induces not only an increase in eye velocity at the timing of target reversal but also a decrease in eye velocity at midcycle immediately before the target begins to slow down^42^. In light of the concept that the FPA controls internal gain such that electrical stimulation to the FPA produces an omnidirectional enhancement in the changes in eye velocity that occur in response to sudden changes in target motion^24^, the changes of eye velocity induced by TMS to the FPA suggest that this frontal area also has a role in the gain control of predictive signals^42^. Importantly, the internal smooth pursuit gain in the FPA is modulated based on motor output in past time sequences^25^. Thus, the control of predictive pursuit based on the history of past trials may involve signals based on working memory via the SEF and internal gain control in the FPA.

Finally, it should be kept in mind that both history effects on predictive pursuit observed in this study were tested in a situation where the target velocity was presented according to the uniform distribution; moreover, the observers had prior knowledge of the range of target velocity. In natural situations, the probability of a future event occurring may be biased in some way, and this rule may change unpredictably. In such a situation, the dependence of past experience is likely to be more complex than a linear relationship. A recent study has demonstrated the effect of previous trials on the control of predictive pursuit in a volatile environment where the probability bias of the target direction could change at random times^46^. The study concluded that in such situations, Bayesian hierarchical inference provides a better prediction of the observers’ behavioral responses compared to the classical leaky integrator model (equivalent to the weighted averaging strategy)^46^. In future studies, it will be necessary to clarify whether the control of predictive pursuit based on Bayesian inference can be applied to situations other than unpredictability in the target direction, such as unpredictability of the timing and velocity of target motion.

In summary, our findings expand those of previous studies related to predictive pursuit. The results of this study suggest that predictive pursuit systems allow us to track target motion with randomized velocity using weighted averaging of the information of target velocity (stimulus) and motor output (behavior) in past time sequences; however, these historical effects have partially different influences on predictive pursuit.
